# Chemical composition and anti-herpes simplex virus type 1 (HSV-1) activity of extracts from *Cornus canadensis*

**DOI:** 10.1186/s12906-017-1618-2

**Published:** 2017-02-22

**Authors:** Serge Lavoie, Isabelle Côté, André Pichette, Charles Gauthier, Michaël Ouellet, Francine Nagau-Lavoie, Vakhtang Mshvildadze, Jean Legault

**Affiliations:** 1 Laboratoire LASEVE, Département des Sciences Fondamentales, Université du Québec à Chicoutimi, 555 boul. de l’Université, Chicoutimi, Québec G7H 2B1 Canada; 2 INRS-Institut Armand-Frappier, Université du Québec, 531 boul. des Prairies, Laval, Québec H7V 1B7 Canada

**Keywords:** Traditional medicine, Native American, *Cornus canadensis*, HSV-1, hydrolysable tannins, Tellimagrandin I

## Abstract

**Background:**

Many plants of boreal forest of Quebec have been used by Native Americans to treat a variety of microbial infections. However, the antiviral activities of these plants have been seldom evaluated on cellular models to validate their in vitro efficiencies. In this study, *Cornus canadensis* L. (Cornaceae), a plant used in Native American traditional medicine to treat possible antiviral infections, has been selected for further examination.

**Methods:**

The plant was extracted by decoction and infusion with water, water/ethanol 1:1 and ethanol to obtain extracts similar to those used by Native Americans. The effects of the extracts were tested on herpes simplex virus type-1 (HSV-1) using a plaque reduction assay. Moreover, bioassay-guided fractionation was achieved to isolate bioactive compounds.

**Results:**

Water/ethanol 1:1 infusion of *C. canadensis* leaves were the most active extracts to inhibit virus absorption with EC_50_ of about 9 μg mL^−1^, whereas for direct mode, both extraction methods using water or water/ethanol 1:1 as solvent were relatively similar with EC_50_ ranging from 11 to 17 μg mL^−1^. The fractionation led to the identification of active fractions containing hydrolysable tannins. Tellimagrandin I was found the most active compound with an EC_50_ of 2.6 μM for the direct mode and 5.0 μM for the absorption mode.

**Conclusion:**

Altogether, the results presented in this work support the antiviral activity of *Cornus canadensis* used in Native American traditional medicine.

## Background

Herpes simplex virus type-1 (HSV-1) is one of the most common infections in the human population. The prevalence in the world’s population aged between 0 and 49 years old was estimated in 2012 at 3.7 billion people (67%) [1]. HSV-1 is an encapsulated DNA virus of the family *Herpesviridae*. It is responsible for self-limiting infections causing vesicular lesions of the oral (herpes labialis) or genital mucosa (genital herpes) [1]. Outbreaks of HSV are probably triggered by immune deficiency, emotional stress and UV radiation [2]. Despite available treatments, recurrent oral or genital herpes significantly impairs the quality of life [3, 4]. HSV-1 can also infect the cornea and nervous system, thereby causing encephalitis, corneal blindness and peripherical nervous system disorders [5, 6]. Encephalitis caused by HSV-1 is the most sporadic fatal encephalitis worldwide with significant morbidity and mortality. Over seventy percent of childrens with HSV-1 encephalitis die or have permanent neurological impairment even with antiviral therapy [5, 6]. Acyclovir, an antiviral agent, is currently the preferred drug to treat herpes infection. This deoxyguanosine analogue inhibits HSV-1 replication by targeting the viral DNA polymerase [7]. However, acyclovir, and related drugs, can induce resistance and cause side-effects as acute renal ﻿insufficiency and neurotoxicity [8, 9]. Therefore, new antiviral agents active against HSV-1 must be discovered. Interestingly, plants are an important source of biologically active compounds. About 25% of all new active substances discovered between 1981 and 2014 are natural products or are derived from natural products [10]. Moreover, many plant-derived compounds possess an anti-HSV-1 activity [11].

The herpes virus has been present in Native American populations for thousands of years [12]. *Cornus canadensis* was used by Native American to treat some general symptoms caused by HSV-1 infections such as sores (Thompson), pain (Abnaki, Delaware) and fevers (Iroquois, Costanoan) [13]. However, no study has been conducted to evaluate their in vitro antiviral activity. In this report, leaf extracts from *Cornus canadensis* L. (Cornaceae), used by Native Americans in traditional medicine, have been selected to evaluate their anti-HSV-1 potential.

## Methods

### General

Optical rotations were obtained at the sodium D line (589 nm) on a Jasco DIP-360 digital polarimeter. NMR spectra were recorded at 292 K on a Bruker Avance 400 operating at 400.13 MHz for ^1^H and 100.61 MHz for ^13^C and using a 5 mm QNP probe with a z-gradient coil. All spectra were acquired in methanol-*d*_4_ unless otherwise specified and chemical shifts were reported in ppm (*δ*) relative to TMS. Preparative HPLC were first carried out with an Agilent 1100 series on a Zorbax Eclipse XDB-C_18_ column (4.6 × 250 mm, 5 *μ*m) and then scaled-up on a Zorbax PrepHT Eclipse XDB-C_18_ column (21.2 × 250 mm, 7 *μ*m). Low-pressure liquid chromatography (LPLC) was carried out on a Büchi Sepacore flash system consisting of a control unit (C-620), two pump modules (C-605), a UV detector (C-635), and a fraction collector (C-660). Reagent grade dichloromethane (DCM), methanol (MeOH), hexanes (Hex) and ethyl acetate (EtOAc) were purchased from VWR International (Ville Mont-Royal, Québec, Canada) and used without further purification for the extraction and separation of compounds **1**–**19**. The adsorbents used for open column chromatography (CC) were Diaion HP20 (VWR International, Québec, Canada), silica gel Ultra Pure (40–63 *μ*m, Silicycle, Québec, Canada) and C_18_ reversed phase silica gel Ultra Pure (carbon 11%, 40–63 *μ*m, Silicycle, Québec, Canada). TLC was performed on silica gel 60 F_254_ glass plates (250 *μ*m layer thickness, Silicycle, Québec, Canada). Reversed phase TLC was carried out on Merck RP-18 F_254s_ glass plates. The TLC plates were sprayed with 5% aq. H_2_SO_4_ followed by 1% vanillin in ethanol and heated at 110 °C for 5 min. TLC spots were visualized by inspection of the plates under visible light [14].

### Plant material

All plant specimens were harvested between May and July 2008, or in June 2012, in the “Forêt d’Enseignement et de Recherche Simoncouche” of the *Réserve Faunique des Laurentides*, Québec, Canada (48° 14′ 40″ N, 71° 15′ 15″ W). The plants were identified at the Université du Québec à Chicoutimi by M. Patrick Nadeau. A voucher specimen (QFA0610437) was deposited at the Herbarium Louis-Marie of Université Laval, Québec, Canada. The leaves and the stems were dried at room temperature for one week after which they were grounded and stored at −18 °C until processed.

### Extracts preparation

To mimic the classical uses by the Native Americans, two types of extraction were performed including decoction and infusion. Water, water/ethanol 1:1 and ethanol were used as solvents. Firstly, plant powder (10 g) was boiled in 100 mL of solvent for one hour and the obtained decoction was filtered. The same procedure was repeated three times with the same plant material and the results of the three successive extractions were combined. Secondly, boiling solvent was added to 10 g of powdered plant and mixed for one hour at room temperature. The resulting infusion was filtered and the procedure was repeated three times on the same plant material, as described above. Crude ethanol extracts were concentrated under vacuum and subsequently lyophilized while crude water extracts were only lyophilized.

### Bioassay-guided fractionation

For the bioassay-guided fractionation, a large scale extraction was performed. *C. canadensis* powder (3 594 g) was refluxed in 50% aq. EtOH (43 L). After filtration, the residues were extracted two other times with 2 × 29 L of 50% aq. EtOH. The extraction solutions were combined and concentrated *in vacuo*. The solution was partitioned with CHCl_3_ (4 × 50 L). Both layers were separated and evaporated *in vacuo* yielding a green CHCl_3_ fraction (28.5 g, 0.8%) and a brown aqueous fraction (1098.7 g, 30.6%). The brown gum (550 g) was suspended in water (5 L) and extracted with *n*-BuOH (3 × 2.5 L). Both fractions were evaporated *in vacuo* yielding a brown water fraction (514.4 g, 28.6%) and a brown *n*-BuOH fraction (35.6 g, 2.0%). Each of these extracts (CHCl_3_, *n*-BuOH and H_2_O) were tested for anti-HSV-1 activity.

The *n*-BuOH fraction (35 g) was separated by CC on Diaion with a step gradient of H_2_O and MeOH as follow: H_2_O and MeOH 10% afforded fractions F1 (6.2 g) and F2 (4.6 g), MeOH 30 to 50% afforded fractions F3 (2.4 g) and F4 (8.8 g), MeOH 80% afforded fraction F5 (6.9 g) and MeOH 100% afforded fraction F6 (2.0 g). Fraction F4 was separated by LPLC on silica gel with DCM-MeOH-H_2_O (200:48:7 → 40:48:7) as the eluent followed by MeOH containing 2% acetic acid. The resulting fractions were gathered according to their TLC profiles providing seven fractions: F4.1 (361 mg), F4.2 (548 mg), F4.3 (399 mg), F4.4 (1079 mg), F4.5 (2312 mg), F4.6 (1121 mg) and F4.7 (2207 mg). Fraction F5 was separated by CC on silica gel with DCM-MeOH (6:1 → 0:1) affording ten fractions: F5.1 (35 mg), F5.2 (66 mg), F5.3 (189 mg), F5.4 (388 mg), F5.5 (425 mg), F5.6 (1099 mg), F5.7 (1047 mg), F5.8 (433 mg), F5.9 (817 mg) and F5.10 (2602 mg). Fraction 4.1 was purified by preparative HPLC (H_2_O-CH_3_CN, 9:1 → 7:3 in 30 min) affording compound **8** (4.2 mg) and **10** (0.7 mg). Fraction F4.4 was purified by preparative HPLC (H_2_O-CH_3_CN, 19:1 → 16:4 in 40 min) affording compound **2** (1.7 mg), **3** (3.2 mg), **4** (5.3 mg) and **6** (1.2 mg). Fraction F4.5 was purified by preparative HPLC (H_2_O-CH_3_CN, 19:1 → 16:4 in 40 min) affording compound **1** (1.0 mg), **4** (4.9 mg), **5** (9.3 mg), **6** (5.0 mg), **7** (1.1 mg), and **9** (15.5 mg). Fraction 5.3 was purified by preparative HPLC (H_2_O-CH_3_CN, 9:1 → 6:4 in 30 min) affording compound **8** (13.5 mg) and **18** (3.3 mg). Fraction 5.4 was analyzed by analytical HPLC (H_2_O-CH_3_CN-HCOOH, 950:50:1 → 800:200:1 in 20 min) and was shown to contain pure **11**. Fraction 5.5 was purified by preparative HPLC (H_2_O-CH_3_CN, 8:2 for 5 min then 8:2 → 7:3 in 20 min) yielding a mixture of compounds **16** and **19** (1.7 mg), and pure **13** (11.3 mg), **15** (2.1 mg), and **17** (2.0 mg). Finally, fraction 5.6 was purified by preparative HPLC (H_2_O-CH_3_CN, 9:1 → 7:3 in 30 min) yielding compound **10** (27.7 mg), **11** (1.3 mg), **12** (7.7 mg), **13** (2.8 mg), **14** (3.0 mg), and **15** (2.5 mg). All of these isolates were identified as: 1,6-di-*O*-galloyl-*β*-d-glucopyranose (**1**) [15], 1,2,3-tri-*O*-galloyl-*β*-d-glucopyranose (**2**) [16], 1,2,6-tri-*O*-galloyl-*β*-d-glucopyranose (**3**) [17], 1,2,3,6-tetra-*O*-galloyl-*β*-d-glucopyranose (**4**) [15], 1,2,3,4,6-penta-*O*-galloyl-*β*-d-glucopyranose (**5**) [18], tellimagrandin I (**6**), tellimagrandin II (**7**) [19], ethyl gallate (**8**) [20], caffeic acid (**9**) [21], astragalin (**10**), isoquercetin (**11**) [22], trifolin (**12**) [23], kaempferol 3-*O*-*β*-d-xylopyranoside (**13**) [24], reinutrin (**14**) [25], juglanin (**15**), avicularin (**16**) [26], juglalin (**17**) [27], benzyl 2-*O*-*β*-glucopyranosyl-2,6- hydroxybenzoate (**18**) [28] and byzantionoside B (**19**) [29].

### Characterization of isolated compounds

1,6-Di-*O*-galloyl-*β*-d-glucopyranose (**1**): Brown amorphous solid; ^1^H NMR (400 MHz, CD_3_OD) *δ*: 7.12 (2H, s, H-2,6^I^), 7.07 (2H, s, H-2,6^VI^), 5.68 (1H, d, *J* = 6.4 Hz, H-1), 4.54 (1H, br d, *J* = 12.0 Hz, H-6a), 4.39 (1 H, dd, *J* = 12.0, 4.9 Hz, H-6b), 3.71 (1H, m, H-5), 3.51 (3H, m, H-2, H-3, H-4); ^13^C NMR (100 MHz, CD_3_OD) *δ*: 168.34 (s, C-7^VI^), 167.03 (s, C-7^I^), 146.56 (s, C-3,5^VI^), 146.52 (s, C-3,5^I^), 140.51 (s, C-4^I^), 139.93 (s, C-4^VI^), 121.31 (s, C-1^VI^), 120.61 (s, C-1^I^), 110.61 (d, C-2,6^I^), 110.22 (d, C-2,6^VI^), 95.98 (d, C-1), 78.07 (d, C-3), 76.51 (d, C-5), 74.14 (d, C-2), 71.23 (d, C-4), 64.47 (t, C-6).

1,2,3-Tri-*O*-galloyl-*β*-d-glucopyranose (**2**): Brown amorphous solid; [*α*]_D_^25^ +33.3° (*c* = 0.04, MeOH); ^1^H NMR (400 MHz, CD_3_OD) *δ*: 7.03 (2H, s, H-2,6^III^), 7.02 (2H, s, H-2,6^I^), 6.91 (2H, s, H-2,6^II^), 6.05 (1H, d, *J* = 8.3 Hz, H-1), 5.53 (1H, t, *J* = 9.5 Hz, H-3), 5.41 (1H, dd, *J* = 9.9, 8.2 Hz, H-2), 3.92 (1H, br d, *J* = 12.9 Hz, H-6a), 3.88 (1H, t, *J* = 9.7 Hz, H-4), 3.80 (1H, dd, *J* = 12.2, 4.6 Hz, H-6b), 3.69 (1H, m, H-5); ^13^C NMR (100 MHz, CD_3_OD) *δ*: 167.83 (s, C-7^III^), 167.20 (s, C-7^II^), 166.42 (s, C-7^I^), 146.56 (s, C-4^I^), 146.39 (s, C-4^III^), 146.36 (s, C-4^II^), 140.68 (s, C-3,5^I^), 140.20 (s, C-3,5^II^), 139.99 (s, C-3,5^III^), 121.09 (s, C-1^III^), 120.51 (s, C-1^II^), 120.00 (s, C-1^I^), 110.56 (d, C-2,6^I^), 110.43 (d, C-2,6^III^), 110.39 (d, C-2,6^II^), 93.92 (d, C-1), 79.07 (d, C-5), 76.79 (d, C-3), 72.45 (d, C-2), 69.35 (d, C-4), 61.89 (t, C-6).

1,2,6-Tri-*O*-galloyl-*β*-d-glucopyranose (**3**): Brown amorphous solid; [*α*]_D_^25^ −80.7° (*c* = 0.09, MeOH); ^1^H NMR (400 MHz, CD_3_OD) *δ*: 7.11 (2H, s, H-2,6^VI^), 7.05 (2H, s, H-2,6^II^), 7.01 (2H, s, H-2,6^I^), 5.93 (1H, d, *J* = 8.4 Hz, H-1), 5.22 (1H, t, *J* = 9.0 Hz, H-2), 4.57 (1H, br d, *J* = 12.0 Hz, H-6a), 4.47 (1H, dd, *J* = 12.1, 4.6 Hz, H-6b), 3.84 (1H, m, H-5), 3.83 (1H, m, H-3), 3.67 (1H, t, *J* = 9.3 Hz, H-4); ^13^C NMR (100 MHz, CD_3_OD) *δ*: 168.30 (s, C-7^VI^), 167.62 (s, C-7^II^), 166.54 (s, C-7^I^), 146.54 (s, C-4^VI^), 146.51 (s, C-4^I^), 146.45 (s, C-4^II^), 140.63 (s, C-3,5^I^), 140.08 (s, C-3,5^II^), 139.95 (s, C-3,5^VI^), 121.30 (s, C-1^VI^), 121.08 (s, C-1^II^), 120.02 (s, C-1^I^), 110.60 (d, C-2,6^I^), 110.41 (d, C-2,6^II^), 110.24 (d, C-2,6^VI^), 94.15 (d, C-1), 76.65 (d, C-5), 76.00 (d, C-3), 74.31 (d, C-2), 71.40 (d, C-4), 64.26 (t, C-6).

1,2,3,6-Tetra-*O*-galloyl-*β*-d-glucopyranose (**4**): Brown amorphous solid; [*α*]_D_^25^ +37.4° (*c* = 0.18, MeOH); ^1^H NMR (400 MHz, CD_3_OD) *δ*: 7.13 (2H, s, H-2,6^VI^), 7.04 (2H, s, H-2,6^III^), 7.03 (2H, s, H-2,6^I^), 6.94 (2H, s, H-2,6^II^), 6.11 (1H, d, *J* = 8.3 Hz, H-1), 5.59 (1H, dd, *J* = 9.6, 9.0 Hz, H-3), 5.45 (1H, dd, *J* = 9.9, 8.3 Hz, H-2), 4.62 (1H, dd, *J* = 12.4, 1.9 Hz, H-6a), 4.53 (1H, dd, *J* = 12.1, 4.3 Hz, H-6b), 4.03 (1H, ddd, *J* = 10.0, 4.3, 1.9 Hz, H-5), 3.97 (1H, dd, *J* = 9.7, 8.8 Hz, H-4); ^13^C NMR (100 MHz, CD_3_OD) *δ*: 168.19 (s, C-7^VI^), 167.74 (s, C-7^III^), 167.20 (s, C-7^II^), 166.34 (s, C-7^I^), 146.54 (s, C-4^VI^), 146.54 (s, C-4^I^), 146.38 (s, C-4^III^), 146.35 (s, C-4^II^), 140.75 (s, C-3,5^I^), 140.24 (s, C-3,5^II^), 140.03 (s, C-3,5^III^), 140.00 (s, C-3,5^VI^), 121.23 (s, C-1^VI^), 120.98 (s, C-1^III^), 120.41 (s, C-1^II^), 119.84 (s, C-1^I^), 110.61 (d, C-2,6^I^), 110.43 (d, C-2,6^III^), 110.40 (d, C-2,6^II^), 110.24 (d, C-2,6^VI^), 93.93 (d, C-1), 76.65 (d, C-5), 76.49 (d, C-3), 72.39 (d, C-2), 69.66 (d, C-4), 64.00 (t, C-6).

1,2,3,4,6-Penta-*O*-galloyl-*β*-d-glucopyranose (**5**): Brown amorphous solid; [*α*]_D_^25^ +21.8° (*c* = 0.29, MeOH); ^1^H NMR (400 MHz, CD_3_OD) *δ*: 7.12 (2H, s, H-2^VI^), 7.06 (2H, s, H-2^I^), 6.98 (2H, s, H-2^IV^), 6.95 (2H, s, H-2^II^), 6.90 (2H, s, H-2^III^), 6.25 (1H, d, *J* = 8.3 Hz, H-1), 5.92 (1H, t, *J* = 9.7 Hz, H-3), 5.63 (1H, t, *J* = 9.7 Hz, H-4), 5.59 (1H, dd, *J* = 9.8, 8.4 Hz, H-2), 4.52 (1H, br d, *J* = 11.1 Hz, H-6a), 4.42 (1H, m, H-5), 4.39 (1H, m, H-6b); ^13^C NMR (100 MHz, CD_3_OD) *δ*: 167.94 (C-7^VI^), 167.31 (C-7^III^), 167.03 (C-7^II^), 166.93 (C-7^IV^), 166.23 (C-7^I^), 146.57 (C-3^I^), 146.49 (C-3^VI^), 146.46 (C-3^IV^), 146.39 (C-3^II^), 146.30 (C-3^III^), 140.78 (C-4^I^), 140.37 (C-4^IV^), 140.32 (C-4^II^), 140.14 (C-4^III^), 140.02 (C-4^VI^), 121.05 (C-1^VI^), 120.37 (C-1^III^), 120.25 (C-1^II^), 120.21 (C-1^IV^), 119.73 (C-1^I^), 110.62 (C-2^I^), 110.47 (C-2^IV^), 110.41 (C-2^II^), 110.38 (C-2^III^), 110.34 (C-2^VI^), 93.83 (C-1), 74.44 (C-5), 74.13 (C-3), 72.20 (C-2), 69.80 (C-4), 63.13 (C-6).

Tellimagrandin I (2,3-di-*O*-galloyl-4,6-hexahydroxydiphenoyl-*β*-d-glucopyranose, **6**): Brown amorphous solid; [*α*]_D_^25^ +70.5° (*c* = 0.16, MeOH); ^1^H NMR (400 MHz, CD_3_OD) *δ*: *α*-anomer (60%): 7.01 (2H, s, H-2,6^II^), 6.92 (2H, s, H-2,6^III^), 6.60 (1H, s, H-2^VI^), 6.49 (1H, s, H-2^IV^), 5.83 (1H, t, *J* = 10.0 Hz, H-3), 5.49 (1H, d, *J* = 3.8 Hz, H-1), 5.32 (1H, dd, *J* = 12.7, 6.6 Hz, H-6), 5.12 (1H, t, *J* = 9.9 Hz, H-4), 5.09 (1H, dd, *J* = 9.4, 4.5 Hz, H-2), 4.64 (1H, dd, *J* = 10.1, 6.8 Hz, H-5), 3.82 (1H, d, *J* = 12.8 Hz, H-6); *β*-anomer (40%): 6.99 (2H, s, H-2,6^II^), 6.89 (2H, s, H-2,6^III^), 6.60 (1H, s, H-2^VI^), 6.45 (1H, s, H-2^IV^), 5.59 (1H, t, *J* = 9.7 Hz, H-3), 5.35 (1H, dd, *J* = 12.8, 6.6 Hz, H-6), 5.19 (1H, dd, *J* = 9.6, 8.1 Hz, H-2), 5.14 (1H, t, *J* = 9.9 Hz, H-4), 4.96 (1H, d, *J* = 8.1 Hz, H-1), 4.21 (1H, dd, *J* = 9.8, 6.4 Hz, H-5), 3.90 (1H, d, *J* = 13.0 Hz, H-6); ^13^C NMR (100 MHz, CD_3_OD) *δ*: *α* anomer: 169.78 (s, C-7^VI^), 169.32 (s, C-7^IV^), 167.94 (s, C-7^III^), 167.48 (s, C-7^II^), 146.41 (s, C-4^II^), 146.22 (s, C-4^III^), 145.94 (s, C-4^IV^), 145.87 (s, C-4^VI^), 144.82 (2×, s, C-5^IV^ and C-5^VI^), 140.16 (s, C-3,5^II^), 139.93 (s, C-3,5^III^), 137.62 (2×, s, C-3^IV^ and C-3^VI^), 126.36 (s, C-1^IV^), 125.96 (s, C-1^VI^), 120.79 (s, C-1^III^), 120.60 (s, C-1^II^), 116.71 (s, C-6^VI^), 116.42 (s, C-6^IV^), 110.46 (d, C-2,6^III^), 110.42 (d, C-2,6^II^), 108.66 (d, C-2^VI^), 108.26 (d, C-2^IV^), 91.80 (d, C-1), 73.61 (d, C-2), 72.00 (d, C-3), 71.99 (d, C-4), 67.61 (d, C-5), 64.29 (t, C-6); *β* anomer: 169.68 (s, C-7^VI^), 169.24 (s, C-7^IV^), 167.70 (s, C-7^III^), 167.12 (s, C-7^II^), 146.38 (s, C-4^II^), 146.19 (s, C-4^III^), 145.93 (s, C-4^IV^), 145.89 (s, C-4^VI^), 144.82 (2×, s, C-5^IV^ and C-5^VI^), 140.01 (s, C-3,5^II^), 139.95 (s, C-3,5^III^), 137.62 (2×, s, C-3^IV^ and C-3^VI^), 126.32 (s, C-1^IV^), 125.89 (s, C-1^VI^), 120.93 (s, C-1^III^), 120.59 (s, C-1^II^), 116.66 (s, C-6^VI^), 116.46 (s, C-6^IV^), 110.48 (d, C-2,6^III^), 110.37 (d, C-2,6^II^), 108.62 (d, C-2^VI^), 108.24 (d, C-2^IV^), 97.12 (d, C-1), 74.82 (d, C-2), 74.33 (d, C-3), 71.69 (d, C-4), 71.59 (d, C-5), 64.22 (t, C-6).

 Tellimagrandin II (1,2,3-tri-*O*-galloyl-4,6-hexahydroxydiphenoyl-*β*-d-glucopyranose, **7**): Brown amorphous solid; [*α*]_D_^25^ +32.1° (*c* = 0.08, MeOH); ^1^H NMR (400 MHz, CD_3_OD) *δ*: 7.05 (2H, s, H-2,6^I^), 6.95 (2H, s, H-2,6^II^), 6.91 (2H, s, H-2,6^III^), 6.61 (1H, s, H-2^VI^), 6.48 (1H, s, H-2^IV^), 6.11 (1H, d, *J* = 8.3 Hz, H-1), 5.76 (1H, t, *J* = 9.7 Hz, H-3), 5.54 (1H, dd, *J* = 9.3, 8.4 Hz, H-2), 5.39 (1H, dd, *J* = 13.3, 6.5 Hz, H-6a), 5.23 (1H, t, *J* = 9.9 Hz, H-4), 4.43 (1H, dd, *J* = 10.1, 6.4 Hz, H-5), 3.92 (1H, d, *J* = 13.3 Hz, H-6b); ^13^C NMR (100 MHz, CD_3_OD) *δ*: 169.59 (s, C-7^VI^), 169.24 (s, C-7^IV^), 167.57 (s, C-7^III^), 166.92 (s, C-7^II^), 166.19 (s, C-7^I^), 146.62 (s, C-4^I^), 146.43 (s, C-4^II^), 146.27 (s, C-4^III^), 145.86 (s, C-4^IV^), 145.82 (s, C-4^VI^), 145.17 (s, C-5^IV^), 145.15 (s, C-5^VI^), 140.82 (s, C-3,5^I^), 140.33 (s, C-3,5^II^), 140.11 (s, C-3,5^III^), 137.80 (s, C-3^IV^), 137.78 (s, C-3^VI^), 126.24 (s, C-1^IV^), 126.24 (s, C-1^VI^), 120.45 (s, C-1^III^), 120.32 (s, C-1^II^), 119.74 (s, C-1^I^), 116.87 (d, C-6^VI^), 116.69 (d, C-6^IV^), 110.61 (d, C-2,6^I^), 110.53 (d, C-2,6^III^), 110.41 (d, C-2,6^II^), 108.59 (d, C-2^VI^), 108.21 (d, C-2^IV^), 94.2 (d, C-1), 74.07 (d, C-3), 73.64 (d, C-5), 72.47 (d, C-2), 71.28 (d, C-4), 63.73 (t, C-6).

Ethyl gallate (**8**): Brown amorphous solid; ^1^H NMR (400 MHz, CD_3_OD) *δ*: 7.05 (2H, s, H-2,6), 4.27 (2H, q, *J* = 7.1 Hz, H-1′), 1.35 (3 H, t, *J* = 7.1 Hz, H-2′); ^13^C NMR (100 MHz, CD_3_OD) *δ*: 168.58 (s, C-7), 146.50 (s, C-3,5), 139.71 (s, C-4), 121.77 (s, C-1), 110.00 (d, C-2,6), 61.71 (t, C-1′), 14.66 (q, C-2′).

Caffeic acid (**9**): White amorphous solid; ^1^H NMR (400 MHz, CD_3_OD) *δ*: 7.59 (1H, d, *J* = 15.9 Hz, H-7), 7.05 (1H, d, *J* = 2.0 Hz, H-2), 6.95 (1H, dd, *J* = 8.3, 2.0 Hz, H-6), 6.77 (1H, d, *J* = 8.2 Hz, H-5), 6.30 (1H, d, *J* = 15.9 Hz, H-8); ^13^C NMR (100 MHz, CD_3_OD) *δ*: 169.05 (s, C-9), 149.65 (s, C-4), 147.26 (d, C-7), 146.84 (s, C-3), 127.76 (s, C-1), 123.04 (d, C-6), 116.52 (d, C-5), 115.20 (d, C-2), 114.88 (d, C-8).

Astragalin (kaempferol 3-*O*-*β*-d-glucopyranoside, **10**): Yellow amorphous solid; [*α*]_D_^25^ −36.3° (*c* = 0.43, MeOH); ^1^H NMR (400 MHz, CD_3_OD) *δ*: 8.05 (2H, d, *J* = 8.7 Hz, H-2′,6′), 6.88 (2H, d, *J* = 8.8 Hz, H-3′,5′), 6.39 (1H, d, *J* = 2.0 Hz, H-8), 6.19 (1H, d, *J* = 2.0 Hz, H-6), 5.26 (1H, d, *J* = 7.2 Hz, H-1″), 3.69 (1H, dd, *J* = 11.9, 2.3 Hz, H-6″a), 3.53 (1H, dd, *J* = 12.0, 5.5 Hz, H-6″b), 3.43 (1H, m, H-2″), 3.42 (1H, m, H-3″), 3.30 (1H, m, H-4″), 3.20 (1H, ddd, *J* = 9.7, 5.5, 2.3 Hz, H-5″); ^13^C NMR (100 MHz, CD_3_OD) *δ*: 179.53 (s, C-4), 166.21 (s, C-7), 163.11 (s, C-5), 161.61 (s, C-4′), 159.07 (s, C-2), 158.55 (s, C-9), 135.47 (s, C-3), 132.31 (d, C-2′,6′), 122.82 (s, C-1′), 116.10 (d, C-3′,5′), 105.71 (s, C-10), 104.09 (d, C-1″), 99.96 (d, C-6), 94.81 (d, C-8), 78.46 (d, C-5″), 78.07 (d, C-3″), 75.77 (d, C-2″), 71.38 (d, C-4″), 62.65 (t, C-6″).

Isoquercetin (quercetin 3-*O*-*β*-d-glucopyranoside, **11**): Yellow amorphous solid; [*α*]_D_^25^ −24.6° (*c* = 0.02, MeOH); ^1^H NMR (400 MHz, CD_3_OD) *δ*: 7.71 (1H, d, *J* = 2.2 Hz, H-2′), 7.59 (1H, dd, *J* = 8.4, 2.0 Hz, H-6′), 6.86 (1H, d, *J* = 8.5 Hz, H-5′), 6.36 (1H, br s, H-8), 6.18 (1H, d, *J* = 1.8 Hz, H-6), 5.23 (1H, d, *J* = 7.5 Hz, H-1″), 3.71 (1H, dd, *J* = 11.9, 2.3 Hz, H-6″a), 3.57 (1H, dd, *J* = 11.9, 5.2 Hz, H-6″b), 3.47 (1H, t, *J* = 8.2 Hz, H-2″), 3.42 (1H, t, *J* = 8.6 Hz, H-3″), 3.34 (1H, m, H-4″), 3.21 (1H, m, H-5″);

Trifolin (kaempferol 3-*O*-*β*-d-galactopyranoside, **12**): Yellow amorphous solid; [*α*]_D_^25^ −32.4° (*c* = 0.11, MeOH); ^1^H NMR (400 MHz, CD_3_OD) *δ*: 8.09 (2H, d, *J* = 8.7 Hz, H-2′,6′), 6.88 (2H, d, *J* = 8.7 Hz, H-3′,5′), 6.40 (1H, d, *J* = 1.5 Hz, H-8), 6.20 (1H, d, *J* = 1.9 Hz, H-6), 5.14 (1H, d, *J* = 7.8 Hz, H-1″), 3.81 (1H, d, *J* = 3.5 Hz, H-4″), 3.78 (1H, dd, *J* = 9.6, 7.9 Hz, H-2″), 3.62 (1H, dd, *J* = 11.1, 6.1 Hz, H-6″a), 3.52 (1H, m, H-3″), 3.51 (1H, m, H-6″b), 3.43 (1H, t, *J* = 6.1 Hz, H-5″); ^13^C NMR (100 MHz, CD_3_OD) *δ*: 166.40 (s, C-7), 163.10 (s, C-5), 161.65 (s, C-4′), 159.04 (s, C-2), 158.57 (s, C-9), 135.59 (s, C-3), 132.40 (d, C-2′,6′), 122.73 (s, C-1′), 116.14 (d, C-3′,5′), 105.61 (s, C-10), 105.00 (d, C-1″), 100.02 (d, C-6), 94.85 (d, C-8), 77.17 (d, C-5″), 75.07 (d, C-3″), 73.05 (d, C-2″), 70.05 (d, C-4″), 62.01 (t, C-6″).

Kaempferol 3-*O*-*β*-d-xylopyranoside (**13**): Yellow amorphous solid; [*α*]_D_^25^ −63.8° (*c* = 0.18, MeOH); ^1^H NMR (400 MHz, CD_3_OD) *δ*: 8.03 (2H, d, J = 8.8 Hz, H-2′,6′), 6.88 (2H, d, J = 8.8 Hz, H-3′,5′), 6.40 (1H, d, *J* = 2.1 Hz, H-8), 6.20 (1H, d, *J* = 2.0 Hz, H-6), 5.19 (1H, d, *J* = 7.2 Hz, H-1″), 3.77 (1H, dd, *J* = 11.5, 5.1 Hz, H-5″a), 3.48 (1H, m, H-2″), 3.48 (1H, m, H-4″), 3.40 (1H, t, *J* = 8.6 Hz, H-3″), 3.11 (1H, dd, *J* = 11.6, 9.6 Hz, H-5″b); ^13^C NMR (100 MHz, CD_3_OD) *δ*: 179.4 (s, C-4), 166.1 (s, C-7), 163.0 (s, C-5), 161.6 (s, C-4′), 158.9 (s, C-2), 158.4 (s, C-9), 135.3 (s, C-3), 132.2 (d, C-2′,6′), 122.6 (s, C-1′), 116.1 (d, C-3′,5′), 105.6 (s, C-10), 104.6 (d, C-1″), 99.9 (d, C-6), 94.8 (d, C-8), 77.5 (d, C-3″), 75.3 (d, C-2″), 71.0 (d, C-4″), 67.2 (t, C-5″).

Reinutrin (quercetin 3-*O*-*β*-xylopyranoside, **14**): Brown amorphous solid; ^1^H NMR (400 MHz, CD_3_OD) *δ*: 7.60 (1H, m, H-2′), 7.59 (1H, m, H-6′), 6.85 (1H, d, *J* = 8.4 Hz, H-5′), 6.39 (1H, br s, H-8), 6.20 (1H, br s, H-6), 5.18 (1H, d, *J* = 7.2 Hz, H-1″), 3.78 (1H, dd, *J* = 11.6, 5.2 Hz, H-5″a), 3.51 (1H, m, H-2″), 3.50 (1H, m, H-4″), 3.39 (1H, t, *J* = 8.6 Hz, H-5″b), 3.09 (1H, dd, *J* = 11.7, 9.6 Hz, H-5″b). ^13^C NMR (100 MHz, CD_3_OD) *δ*: 166.6 (C-7), 163.1 (C-5), 159.8 (C-2), 158.5 (C-9), 149.9 (C-4′), 146.1 (C-3′), 135.4 (C-3), 123.3 (C-6′), 123.1 (C-1′), 117.2 (C-2′), 116.0 (C-5′), 106.3 (C-10), 105.5 (C-1″), 100.1 (C-6), 94.8 (C-8), 77.6 (C-3″), 75.3 (C-2″), 71.0 (C-4″), 67.3 (C-5″).

Juglanin (kaempferol 3-*O*-*α*-l-arabinofuranoside, **15**): Yellow amorphous solid; [α_D_^25^] −112.9° (*c* = 0.16, MeOH); ^1^H NMR (400 MHz, CD_3_OD) *δ*: 7.96 (2H, d, *J* = 8.5 Hz, H-2′,6′), 6.93 (2H, d, *J* = 8.5 Hz, H-3′,5′), 6.41 (1H, d, *J* = 2.1 Hz, H-8), 6.21 (1H, d, *J* = 2.1 Hz, H-6), 5.49 (1H, s, H-1″), 4.33 (1H, d, *J* = 3.0 Hz, H-2″), 3.91 (1H, dd, *J* = 5.2, 3.1 Hz, H-3″), 3.80 (1H, dd, *J* = 9.3, 4.7 Hz, H-4″), 3.49 (2H, d, *J* = 4.2 Hz, H-5″); ^13^C NMR (100 MHz, CD_3_OD) *δ*: 179.95 (s, C-4), 166.04 (s, C-7), 163.12 (s, C-5), 161.60 (s, C-4′), 159.42 (s, C-2), 158.59 (s, C-9), 134.96 (s, C-3), 132.02 (d, C-2′,6′), 122.80 (s, C-1′), 116.54 (d, C-3′,5′), 109.65 (d, C-1″), 105.69 (s, C-10), 99.90 (d, C-6), 94.82 (d, C-8), 88.03 (d, C-4″), 83.39 (d, C-2″), 78.66 (d, C-3″), 62.55 (t, C-5″).

Avicularin (quercetin 3-*O*-*α*-arabinofuranoside, **16**): Orange amorphous solid; ^1^H NMR (400 MHz, CD_3_OD) *δ*: 7.53 (1H, d, *J* = 2.1 Hz, H-2′), 7.50 (1H, dd, *J* = 8.3, 2.1 Hz, H-6′), 6.91 (1H, d, *J* = 8.3 Hz, H-5′), 6.40 (1H, d, *J* = 2.1 Hz, H-8), 6.22 (1H, d, *J* = 1.9 Hz, H-6), 5.47 (1H, s, H-1″), 4.33 (1H, s, H-2″), 3.92 (1H, dd, *J* = 5.2, 2.9 Hz, H-3″), 3.86 (1H, m, H-4″), 3.50 (2H, m, H-5″); ^13^C NMR (100 MHz, CD_3_OD) *δ*: 180.02 (s, C-4), 166.20 (s, C-7), 163.12 (s, C-5), 159.38 (s, C-2), 158.62 (s, C-9), 149.90 (s, C-4′), 146.41 (s, C-3′), 134.94 (s, C-3), 123.13 (s, C-1′), 122.99 (d, C-6′), 116.86 (d, C-2′), 116.47 (d, C-5′), 109.55 (d, C-1″), 105.62 (s, C-10), 99.95 (d, C-6), 94.83 (d, C-8), 88.05 (d, C-4″), 83.35 (d, C-2″), 78.73 (d, C-3″), 62.57 (t, C-5″).

Juglalin (kaempferol 3-*O*-*α*-arabinopyranoside, **17**): Yellow amorphous solid; ^1^H NMR (400 MHz, CD_3_OD) *δ*: 8.06 (2H, d, *J* = 8.6 Hz, H-2′,6′), 6.89 (2H, d, *J* = 8.5 Hz, H-3′,5′), 6.40 (1H, d, *J* = 2.0 Hz, H-8), 6.20 (1H, d, *J* = 1.9 Hz, H-6), 5.14 (1H, d, *J* = 6.4 Hz, H-1″), 3.89 (1H, dd, *J* = 8.2, 6.3 Hz, H-2″), 3.78 (1H, m, H-4″), 3.77 (1H, m, H-5″a), 3.63 (1H, m, H-3″), 3.40 (1H, dd, *J* = 13.5, 3.1 Hz, H-5″b); ^13^C NMR (100 MHz, CD_3_OD) *δ*: 179.58 (s, C-4), 166.42 (s, C-7), 163.11 (s, C-5), 161.68 (s, C-4′), 158.84 (s, C-2), 158.53 (s, C-9), 135.58 (s, C-3), 132.30 (d, C-2′,6′), 122.66 (s, C-1′), 116.29 (d, C-3′,5′), 105.60 (s, C-10), 104.41 (d, C-1″), 100.04 (d, C-6), 94.86 (d, C-8), 74.04 (d, C-3″), 72.80 (d, C-2″), 68.97 (d, C-4″), 66.78 (t, C-5″).

Benzyl 2-*O*-*β*-glucopyranosyl-2,6-hydroxybenzoate (**18**): Yellowish oil; [α_D_^25^] −14.8° (*c* = 0.047, MeOH); ^1^H NMR (400 MHz, CD_3_OD) *δ*: 7.50 (2H, d, *J* = 7.4 Hz, H-2′,6′), 7.38 (2H, t, *J* = 7.4 Hz, H-3′,5′), 7.32 (1H, t, *J* = 7.1 Hz, H-4′), 7.27 (1H, t, *J* = 8.3 Hz, H-4), 6.75 (1H, d, *J* = 8.4 Hz, H-3), 6.59 (1H, d, *J* = 8.4 Hz, H-5), 5.38 (2H, s, H-7′), 4.94 (1H, d, *J* = 6.4 Hz, H-1″), 3.86 (1H, br d, *J* = 12.2 Hz, H-6″a), 3.66 (1H, dd, *J* = 12.2, 5.7 Hz, H-6″b), 3.43 (1H, m, H-5″), 3.39 (1H, m, H-2″), 3.39 (1H, m, H-3″), 3.34 (1 H, m, H-4″); ^13^C NMR (100 MHz, CD_3_OD) *δ*: 170.00 (s, C-7), 159.80 (s, C-6), 158.25 (s, C-2), 137.40 (s, C-1′), 134.04 (d, C-4), 129.55 (d, C-3′,5′), 129.22 (2×, d, C-2′,6′, C-4′), 111.57 (d, C-5), 110.87 (s, C-1), 107.79 (d, C-3), 102.75 (d, C-1″), 78.34 (d, C-3″), 77.99 (d, C-5″), 74.93 (d, C-2″), 71.25 (d, C-4″), 68.19 (t, C-7′), 62.54 (t, C-6″).

Byzantionoside B (**19**): Orange amorphous solid; ^1^H NMR (400 MHz, CD_3_OD) *δ*: 5.81 (1H, s, H-4), 4.33 (1H, d, *J* = 8.0 Hz, H-1′), 3.87 (1H, m, H-9), 3.86 (1H, m, H-6′a), 3.65 (1H, m, H-6′b), 3.35 (1H, m, H-3′), 3.26 (1H, m, H-4′), 3.26 (1H, m, H-5′), 3.14 (1H, dd, *J* = 9.1, 7.8 Hz, H-2′), 2.47 (1H, d, *J* = 17.4 Hz, H-2), 2.05 (3H, d, *J* = 1.3 Hz, H-13), 1.98 (1H, m, H-6), 1.97 (1H, m, H-2), 1.95 (1H, m, H-7), 1.63 (2H, m, H-8), 1.51 (1H, m, H-7), 1.19 (3H, d, *J* = 6.2 Hz, H-10), 1.10 (3H, s, H-12), 1.01 (3H, s, H-11); ^13^C NMR (100 MHz, CD_3_OD) *δ*: 202.48 (s, C-3), 170.17 (s, C-5), 125.41 (d, C-4), 102.15 (d, C-1′), 78.20 (d, C-3′), 77.93 (d, C-5′), 75.54 (d, C-9), 75.20 (d, C-2′), 71.88 (d, C-4′), 62.95 (t, C-6′), 52.43 (d, C-6), 48.12 (t, C-2), 37.84 (t, C-8), 37.36 (s, C-1), 29.10 (q, C-11), 27.56 (q, C-12), 26.87 (t, C-7), 25.01 (q, C-13), 19.91 (q, C-10).

### Cell and virus culture

African green monkey kidney cells (Vero, ATCC CCL-81) were obtained from the American Type Culture Collection (ATCC, Manassas, USA). The Vero cell lines were grown in *Eagle’s minimal essential medium* (MEM) (Mediatech Cellgro, VA) supplemented with 10% fetal bovine serum (FBS; Hyclone, Logan, USA), penicillin (100 IU) and streptomycin (100 *μ*g mL^−1^) (Mediatech Cellgro®). Cells were cultured in a humidified atmosphere at 37 °C in 5% CO_2_. The maintenance medium was MEM supplemented with TPCK trypsin (2 *μ*g mL^−1^), glucose (2 mg mL^−1^), bovine serum albumin (1 mg mL^−1^), penicillin (100 IU) and streptomycin (100 *μ*g mL^−1^). HSV-1 (ATCC VR-733) stocks were propagated in Vero cells. Viruses were stored at −80 °C before further analysis. The virus titer was determined by plaque assay [30].

### Cytotoxicity assay

Exponentially growing Vero cells were plated at a density of 15 × 10^3^ cells per well in 96-well microplates (Costar, Corning Inc.) in 100 *μ*L of culture medium and were allowed to adhere before treatment. Then, 100 *μ*L of increasing concentrations of extract dissolved in aqueous DMSO (Sigma–Aldrich) were added. The final solvent concentration in the culture medium was maintained at 0.25% (v/v) to avoid solvent toxicity. The Vero cells were incubated for 72 h in the absence or in the presence of extracts. Cytotoxicity was assessed using the resazurin reduction test [31]. Fluorescence was measured using an automated 96-well Fluoroskan Ascent Fl TM plate reader (Labsystems) at excitation and emission wavelengths of 530 and 590 nm, respectively. Fluorescence was proportional to the cellular metabolic activity in each well. Cytotoxicity was measured as the concentration of extract inhibiting cell growth by 50% (IC_50_).

### Antiviral activity of extracts, fractions and compounds against HSV-1

Plaque reduction assays were performed with monolayer cultures of Vero cells in 24-well culture plates as described by Kock et al. with some modifications [32]. Briefly, the cell monolayer was infected with the virus (30 pfu/well) and incubated at 37 °C with 5% CO_2_ for one hour. The infected cell monolayer was then washed with PBS and overlaid with an overlapping solution (maintenance medium containing 1% methylcellulose and various concentrations of the indicated compounds). After a 72-hour incubation at 37 °C, the cell monolayer was fixed with 5% paraformaldehyde and stained with 0.8% crystal violet. The lysis plaques were counted and the percentage of inhibition was calculated as [(*P*_*control*_ – *P*_*sample*_)/*P*_*control*_] × 100, where *P*_*control*_ and *P*_*sample*_ refer to the lysis plaque number in the absence and in the presence of the extract, fraction or compound, respectively. The effective concentration inhibiting 50% of the lysis plaques induced by HSV-1 (EC_50_) was also calculated from dose-response curves.

### Determination of the mode of antiviral activity

In order to determine the mode of antiviral activity, Vero cells and HSV-1 were incubated with various samples including crude extracts, fractions and compounds at different stages during viral infection [32]. Acyclovir was used as a positive control. To evaluate the protection mode, Vero cells were first pre-treated one hour at 37 °C with samples before infection with HSV-1. Vero cells and viruses were also incubated for one hour at 37 °C together with samples during the absorption period. Moreover, the effect of the sample was tested during the replication period by adding samples to the overlay medium after infection. Finally, to determine the direct mode of antiviral activity, viruses were incubated with the sample for one hour at 37 °C before infection of Vero cells with HSV-1.

### Statistical analysis

All experiments were performed in triplicates for HSV-1. A one-way ANOVA with a Tukey’s multiple comparison test was performed to compare extracts with the negative control for the antiviral assay. The statistical significance was set at *p* < 0.05.

## Results

The aim of this study was to evaluate the anti-HSV-1 activity of a plant used by the Native Americans in traditional medicine. The plant was selected based on its potential use to treat herpes labialis symptoms such as cold sores, fever and pain [13].

### Extraction yield and cytotoxicity of the extracts on Vero cells

Leaves were extracted by decoction or infusion, using water, water/ethanol 1:1 and ethanol as solvent, to obtain extracts similar to those used by Native Americans. Table 1 presents yields of the six extracts expressed as a percentage of the weight of the crude extract to the raw material. The extraction yields range from 12 to 37%. Regardless of the method, the highest yields were obtained with aqueous systems (30 to 37%) and the lowest ones with pure ethanol (12 to 18%). The decoction method using pure water or ethanol as solvents was more efficient in comparison with the infusion method. In contrast, both methods were relatively similar using a water/ethanol 1:1 solvent. On the other hand, the effect of extracts on Vero cell growth was evaluated to ensure that they were not cytotoxic to cells before testing the antiviral activity. Vero cells were incubated in the presence or in the absence of a growing concentration of extract over three days. The results (Table 1), expressed as the concentration inhibiting 50% of cell growth (IC_50_), show that all leaf extracts were not cytotoxic at concentration below 100 *μ*g mL^−1^ except for the ethanol extract (IC_50_ = 88 *μ*g mL^−1^).

**Table 1:** Extraction yield (%) and cytotoxicity on Vero cells of extracts from leaves of *C. canadensis*

Extraction type	Solvent	Extraction yield (%)^a^	Cytotoxicity (IC_50_)^b^
Decoction	H_2_O	36.5	> 100
	H_2_O/EtOH 1:1	34.1	> 100
	EtOH	18.0	> 100
Infusion	H_2_O	30.1	> 100
	H_2_O/EtOH 1:1	34.3	> 100
	EtOH	12.2	88 ± 18

^a^Percentage of weight of crude extract to raw material (10 g)

^b^Concentration (*μ*g mL^−1^) inhibiting 50% of Vero cells growth

### Antiviral activity of the extracts against herpes simplex virus type-1

The antiviral activity of all leaf extracts from *C. canadensis* was evaluated against herpes simplex virus type-1 (HSV-1). Vero cells and viruses were incubated with extracts at different stages of the viral infection to determine the mode of antiviral activity such as protection, absorption, replication and direct modes [32]. Acyclovir, an antiviral agent inhibiting the replication of HSV-1, was used as a positive control. At a concentration of 0.75 *μ*g mL^−1^, acyclovir completely protects Vero cells against infection (data not shown). Firstly, results presented in Table 2 show that Vero cells pre-treatment for one hour with all extracts (100 *μ*g mL^−1^) did not protect them against infection (protection mode). However, the extracts inhibited 90 to 100% of the lysis plaque formation induced by HSV-1 when viruses were pre-treated with the extracts (direct mode) or when Vero cells and viruses were incubated together with the extracts (absorption mode). Moreover, the extracts of *C. canadensis* leaves protected Vero cells during the replication period with an inhibition of the lysis plaque ranging from 69 to 94% at a concentration of 100 *μ*g mL^−1^. The effects of the ethanol extract obtained using infusion were not tested during the replication period due to the cytotoxicity.

**Table 2:** Anti-HSV-1 activities of leaf extracts from *C. canadensis*

Extraction	Solvent	Bioactivity for each mode of antiviral activity^a^			
		Protection^b^	Absorption^c^	Replication^d^	Direct^e^
Decoction	H_2_O	NA	100 (9 ± 1)	69 ± 11 (> 50)	100 (17 ± 6)
	H_2_O/EtOH 1:1	NA	99 ± 2 (31 ± 5)	94 ± 4 (> 50)	100 (14 ± 4)
	EtOH	NA	90 ± 0 (44 ± 8)	91 ± 16 (> 50)	100 (22 ± 3)
Infusion	H_2_O	NA	99 ± 2 (17 ± 2)	85 ± 7 (> 50)	100 (14 ± 3)
	H_2_O/EtOH 1:1	NA	98 ± 4 (9 ± 3)	82 ± 10 (> 50)	100 (11 ± 2)
	EtOH	NA	100 (40 ± 5)	Tx	100 (28 ± 6)

*NA* Inhibition of lysis plaques < 50% was considered not active, *Tx* Cytotoxic against Vero cells at 100 *μ*g mL^−1^

^a^Inhibitory percentage of lysis plaque induced by HSV-1 at a sample concentration of 100 *μ*g mL^−1^ (*top row*), and effective concentration (*μ*g mL^−1^) inhibiting 50% (EC_50_) of lysis plaque (*bottom row; in parentheses*). Acyclovir was used as positive control with 100% inhibition of lysis plaques at a concentration of 0.75 *μ*g mL^−1^

^b^Vero cells were pretreated with compounds prior infection

^c^Vero cells and viruses were incubated together with compounds during the absorption period

^d^Compounds were added after absorption and during the replication period

^e^Viruses were incubated directly with compounds prior infection of Vero cells

The effective concentration inhibiting 50% of lysis plaques induced by HSV-1 (EC_50_) was determined in order to identify the most active extracts from *C. canadensis*. Growing concentrations of extracts ranging from 1.56 to 100 *μ*g mL^−1^ were tested at different stages of viral infection as previously described. Extracts showing an EC_50_ higher than 50 *μ*g mL^−1^ were considered to be inactive. All extracts were inactive in protection mode as well as during the replication period with EC_50_ > 50 *μ*g mL^−1^. On absorption mode, the most active extracts were obtained using decoction method with water as solvent with an EC_50_ of 9 ± 1 *μ*g mL^−1^ and the infusion method with water/ethanol 1:1 as solvent with an EC_50_ of 9 ± 3 *μ*g mL^−1^. On direct mode, both extraction methods using water or water/ethanol 1:1 as solvent were relatively similar with EC_50_ ranging from 11 to 17 *μ*g mL^−1^.

### Bioassay-guided fractionation

**Fig. 1:**
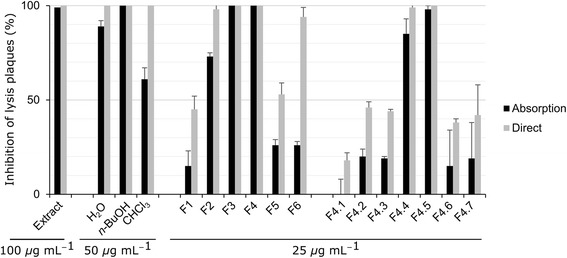
Antiviral activities against HSV-1 of crude extract and fractions obtained from *C. canadensis*. Extract and fractions were incubated with viruses prior infection (*direct mode*) or together with Vero cells and viruses during infection (*absorption mode*). The results are expressed as the percentage of inhibition of the lysis plaques at the indicated concentration

**Fig. 2:**
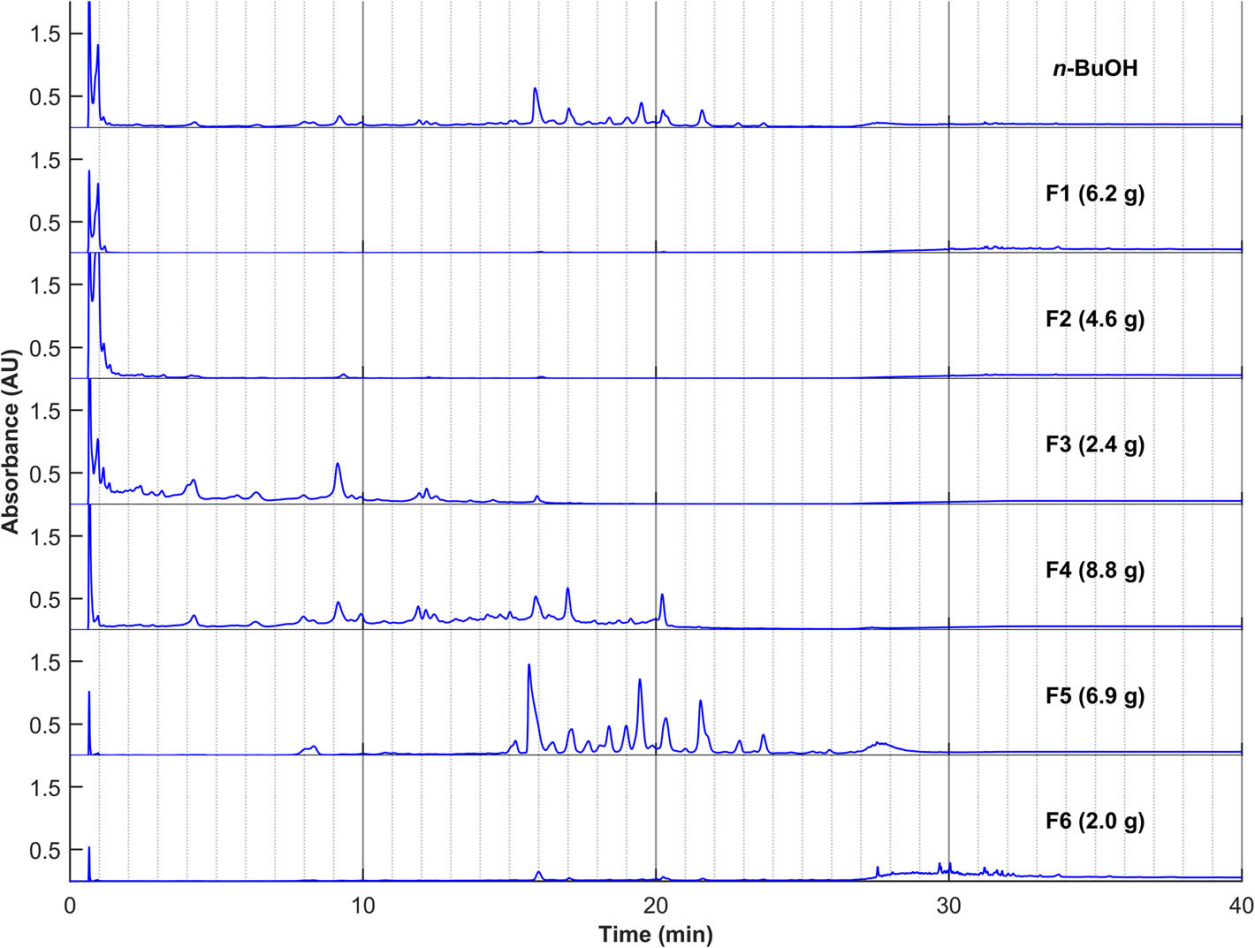
HPLC profiles of fractions F1-F6. In parentheses are the masses obtained after column chromatography. Column: Zorbax Eclipse XDB-C18 column (4.6 × 250 mm, 5 μm); Injection: 10μL at 10 mg mL^−1^; Éluent: H2O + 0.1% HCOOH and CH3CN + 0.1% HCOOH; Program: 5% held for 5 min, 5% to 20% in 20 min, 20% to 90% in 5 min, 90% held for 10 min; Flow: 1 mL min^−1^; Detection: UV 254 ± 50 nm

**Fig. 3:**
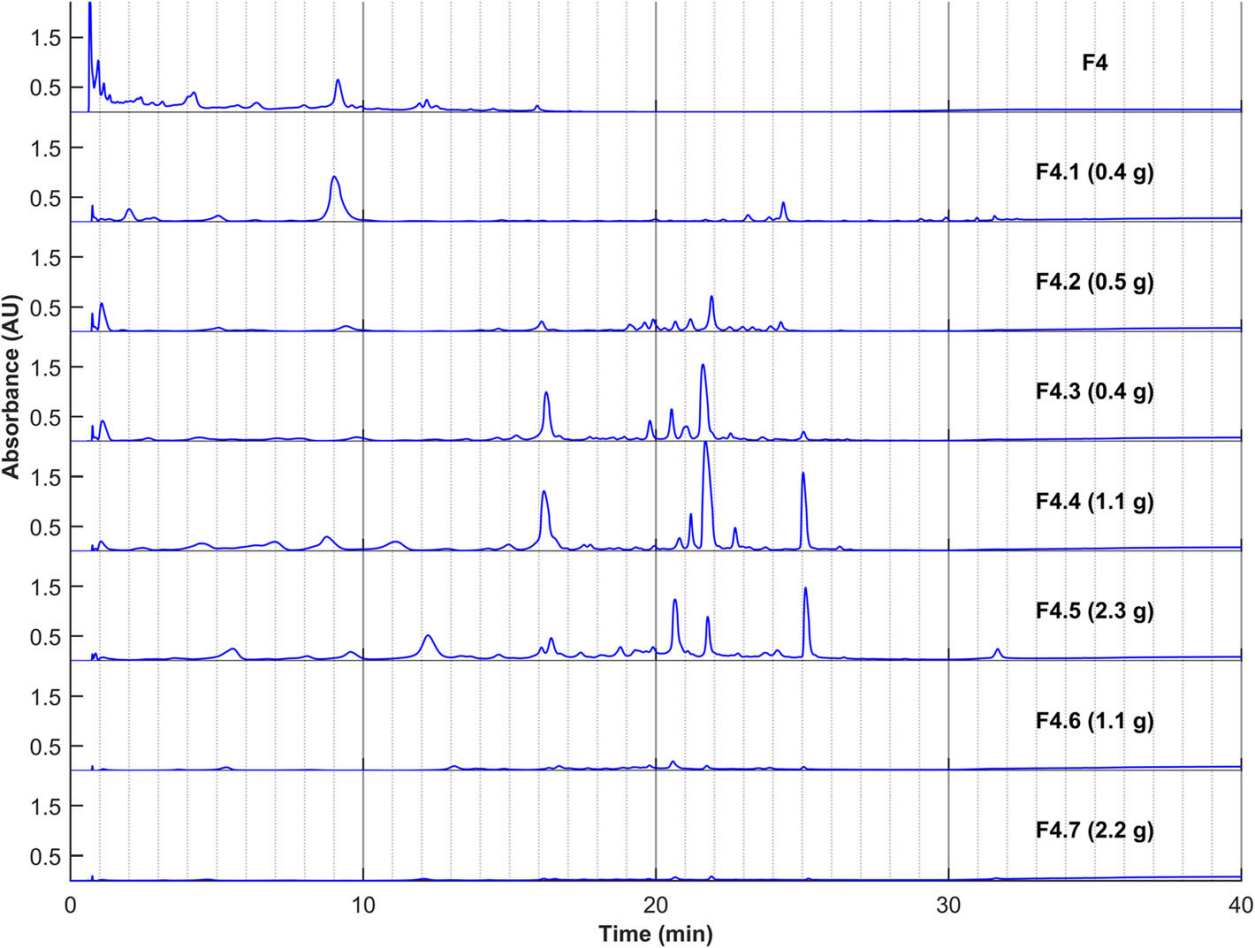
HPLC profiles of fractions F4.1-F4.7. In parentheses are the masses obtained after column chromatography. Column: Zorbax Eclipse XDB-C18 column (4.6 × 250 mm, 5 μm); Injection: 10μL at 10 mg mL^−1^; Eluent: H2O + 0.1% HCOOH and CH3CN + 0.1% HCOOH; Program: 5% held for 5 min, 5% to 20% in 20 min, 20% to 90% in 5 min, 90% held for 10 min; Flow: 1 mL min^−1^; Detection: UV 254 ± 50 nm

A bioassay-guided fractionation was undertaken in order to identify the bioactive components of the extract. For this, a large scale decoction was accomplished from fresh *C. canadensis* leaves. The crude extract was then partitioned by liquid-liquid separation between water, *n*-butanol, and chloroform. The resulting fractions were screened for their anti-HSV-1 activity. The *n*-BuOH fraction showed a complete inhibition of lysis plaques at a concentration of 50 *μ*g mL^−1^ in both the absorption and direct modes and was thus chosen for further fractionations (Fig. 1). Open column chromatography on Diaion® stationary phase was carried out with a gradient of water/methanol as the eluent. The six resulting fractions (F1–F6) were tested against HSV-1 (Fig. 1). Fractions F3 and F4 were the most active toward both modes of activity when tested at 25 *μ*g mL^−1^. Their HPLC profiles (Fig. 2) were almost identical, therefore the most abundant fraction (F4) was chosen for the next step. Silica gel was selected as an adsorbent for low-pressure liquid chromatography using a gradient of dichloromethane and methanol as the eluent. Seven fractions were obtained (F4.1-F4.7), analyzed by HPLC (Fig. 3), and evaluated for their antiviral activity (Fig. 1). The most active fractions (F4.4 and F4.5) were submitted to preparative HPLC yielding seven hydrolysable tannins (**1**–**7**). On the other hand, Fraction F5 was shown to be barely active toward HSV-1, either in the direct or absorption modes (Fig. 1). However, according to TLC, this fraction was rich in secondary metabolites and was thus further purified by silica gel chromatographies followed by reversed-phase preparative HPLC yielding 12 other compounds (**8**–**19**).

**Fig. 4:**
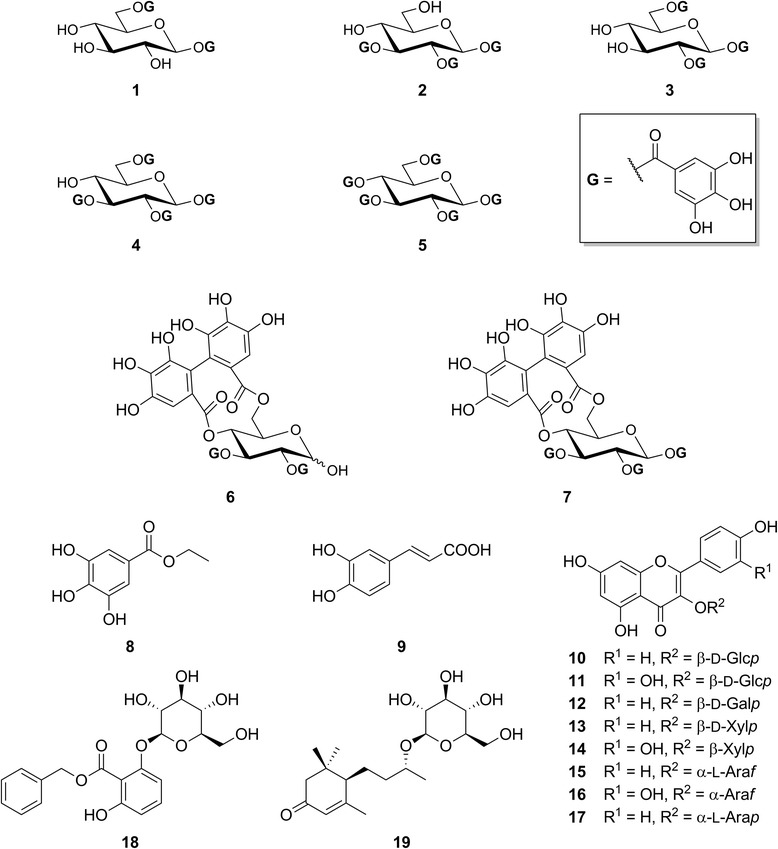
Structures of the isolated compounds from *Cornus canadensis*

The structures of all the isolated compounds were established on the basis of extensive spectroscopic analyses, including 1D and 2D NMR (^1^H–^1^H COSY, HSQC, and HMBC), and by comparison of their respective spectral data with those reported in the literature (Fig. 4). They were identified as: 1,6-di-*O*-galloyl-*β*-d-glucopyranose (**1**), 1,2,3-tri-*O*-galloyl-*β*-d-glucopyranose (**2**), 1,2,6-tri-*O*-galloyl-*β*-d-glucopyranose (**3**), 1,2,3,6-tetra-*O*-galloyl-*β*-d-glucopyranose (**4**), 1,2,3,4,6-penta-*O*-galloyl-*β*-d-glucopyranose (**5**), tellimagrandin I (**6**), tellimagrandin II (**7**), ethyl gallate (**8**), caffeic acid (**9**), astragalin (**10**), isoquercetin (**11**), trifolin (**12**), kaempferol 3-*O*-*β*-d-xylopyranoside (**13**), reinutrin (**14**), juglanin (**15**), avicularin (**16**), juglalin (**17**), benzyl 2-*O*-*β*-glucopyranosyl-2,6-hydroxybenzoate (**18**) and byzantionoside B (**19**).

### Antiviral activity of the isolated compounds

Hydrolysable tannins (**4**–**7**) isolated from the most active fraction (F4) were tested for their anti-HSV-1 activity using a similar protocol as previously described for the extracts and fractions. Growing concentrations of compounds, ranging from 1.56 to 25 *μ*g mL^−1^, were tested in order to evaluate the effective concentration inhibiting 50% of lysis plaques induced by HSV-1 (EC_50_). The results presented in Table 3 showed that the most active compound was tellimagrandin I (**6**) with EC_50_ of 5.0 ± 0.2 and 2.6 ± 0.1 *μ*M for the absorption and direct modes, respectively. The other compounds showed EC_50_ ranging between 7 and 12 *μ*M.

**Table 3:** Anti-HSV-1 activities of isolated compounds (**4-7**) at different stages of viral infection

Compounds	EC_50_ for each mode of antiviral activity (*μ*M)^a^			
	Protection^b^	Absorption^c^	Replication^d^	Direct^e^
4	> 25	11 ± 3	> 25	7 ± 4
5	> 25	12 ± 4	> 25	10 ± 2
6	> 25	5.0 ± 0.2	> 25	2.6 ± 0.1
7	> 25	11 ± 3	> 25	7 ± 1

^a^Effective concentration inhibiting 50% of lysis plaques induced by HSV-1 at different modes of antiviral activity. Acyclovir was used as positive control with 100% inhibition of lysis plaques at a concentration of 3 *μ*M

^b^Vero cells were pretreated with compounds prior infection

^c^Vero cells and viruses were incubated together with compounds during absorption period

^d^Compounds were added after absorption and during the replication period

^e^Viruses were incubated directly with compounds prior infection of Vero cells

## Discussion

*C. canadensis* was used in Native American traditional medicine to treat possible viral infections. The results showed that the hydroalcoholic extract obtained from the infusion of *C. canadensis* acts directly on the virus and also inhibits its absorption by host cells. Some studies aiming to determine the chemical composition of *C. canadensis* showed the presence of iridoids, but no antiviral activity for these compounds was reported [33]. Moreover, seven other iridoids were tested by Bermejo and co-workers against HSV-1 and none of these compounds were found active against this virus [34]. On the other hand, some flavonoids, previously described in *C. canadensis* extracts [35], are also known for their antiviral activity against HSV-1 [36]. For instance, quercetin is known for its virucidal action and its direct inactivation of HSV-1 [37]. However, in the present study, flavonoids were identified from scarcely active fractions. After intensive bioassay-guided fractionation, hydrolysable tannins were finally identified as good candidate compounds responsible for the bioactivity. It is noteworthy that the activity was higher in the direct mode rather than in the absorption mode. These results are consistent with those obtained for the same compounds in other works [38–41] with the exception of tellimagrandin I (**6**), which has been reported more active in another study with an EC_50_ of 0.046 *μ*M [42]. However, this difference could be explained by the use of different host cell lines and virus strains.

## Conclusions

*C. canadensis*, a plant used by Native Americans in traditional medicine to treat possible viral infection, was investigated for its anti-HSV-1 activity. The results reported in this study showed that the hydroalcoholic extract obtained from leaves of *C. canadensis* acts directly on HSV-1, but also inhibits the absorption of the virus by the host cells. Hydrolysable tannins were isolated from the most active fraction and shown to be good candidate compounds responsible for the anti-HSV-1 activity. In a near future, the mechanism of action of the extract and of the bioactive compounds will be determined.
